# The Effect of a Novel Dynamic Hamstring Brace on Muscle and Athletic Performance Tests among Young Basketball Players

**DOI:** 10.5114/jhk/209073

**Published:** 2025-11-19

**Authors:** Roni Gottlieb, Shai Greenberg

**Affiliations:** 1 School of Human Movement and Sport Sciences, Levinsky-Wingate Academic College (Wingate Campus), Netanya, Israel.

**Keywords:** team sports, young athletes, external support, hamstring injuries, injury prevention

## Abstract

A hamstring injury is the most incurred musculoskeletal injury in sports, with high recurrence rates. This study evaluated a novel dynamic hamstring brace on the athletic performance of young male basketball players. The study included 13 healthy adolescent basketball players (aged M = 14 ± 0.6 yrs) who underwent two sets of six performance tests on two different days. Participants wore the dynamic brace for one set of tests (research) and then performed the same tests without the brace (control). Isolated hamstring tests included the end-range hamstring-strength test and the single-leg bridge test. Performance tests included horizontal explosive 10-m and 20-m sprints, vertical explosive counter-movement jumps, and squat jump tests. The findings indicate improved outcomes when wearing the dynamic brace compared to the control condition. A significant increase was seen in the single-leg bridge test (M = 30 ± 4 and 23.5 ± 5 repetitions, respectively, p < 0.01), while a significant decrease was observed in the 10-m sprint test (M = 2.01 ± 0.1 and 1.88 ± 0.1 s, respectively, p < 0.05). No significant differences were found in other tests. In conclusion, the novel brace presented in this study could reduce hamstring injuries, with little impact on athletic performance.

## Introduction

The hamstring is a bi-articular muscle that is activated during high-intensity athletic maneuvers. Yet hamstring injuries are common in sports, with about 1.2–4.0 injuries being incurred for every 1,000 hours of athletic activity (Diemer et al., 2021; Maniar et al., 2023). In soccer, about 22% of all players incur hamstring injuries each season, resulting in an average of 24-days downtime for the player, as well as high costs for the team (Bodendorfer et al., 2023; Green et al., 2020).

An epidemiological study that was conducted among professional soccer players over eight seasons (2014/15 to 2021/22) found that the incidence of hamstring injuries during training and matches had increased significantly over the years, from 12% to 24%, respectively. Moreover, the proportion of all injury-related absences due to hamstring injuries was found to have increased from 10% in 2001/02 to 20% in 2021/22. Finally, almost 20% of all reported hamstring injuries were recurring ones, with over two-thirds occurring within two months of the athlete returning to the field after recovery (Ekstrand et al., 2023a).

As a result, a growing body of research can be seen on hamstring injuries, with the aim of identifying risk factors and developing both prevention and rehabilitation programs (yet with limited success), such as core muscle strengthening (Mendiguchia et al., 2021), hamstring strengthening (Opar et al., 2021), physical-load management (Chebbi et al., 2022), explosive and agility training (Timmins et al., 2021) and passive therapy (Kamandulis et al., 2024).

One main obstacle in such research is the difficulty in identifying the underlying biomechanical mechanisms that are related to such injuries. Danielsson et al. (2020), for example, suggest that the hamstring is most susceptible to injury during active lengthening, which occurs during the late swing phase of the running gait cycle. Mann and Sprague (1980), on the other hand, propose that hamstring injuries occur during the initial stance phase, due to the large opposing forces that are placed on the athlete’s body. It is agreed, however, that most hamstring injuries occur when sprinting, when the hip joint is extended and the knee joint is flexed to 15–30° (i.e., eccentric extension).

Another obstacle for predicting hamstring injuries is the fatigue status of athletes. Clinical observations of elite soccer players (Ekstrand et al., 2023b) suggest a strong correlation between fatigue and hamstring injuries. Laboratory investigations further support this association, demonstrating that hamstring fatigue protocols impair rapid hamstring contraction relative to the quadriceps (Zhang et al., 2021). Additionally, hamstring fatigue has been shown to alter sprint kinematics, leading to biomechanical changes that increase the muscle workload during acceleration (Hegyi et al., 2025; Wilmes et al., 2021).

A key challenge in identifying the underlying causes of hamstring injuries is the lack of reliable physical tests for assessing athletes at risk or determining their readiness to return to full sports participation.

In related studies, tests include isokinetic strength testing (Pieters et al., 2020) and overall vertical and horizontal athletic performance (Huygaerts et al., 2020; Zabaloy et al., 2025). One significant test for predicting hamstring injuries is a single-leg bridge test (SLBT) (Freckleton et al., 2011, 2014; Mahnič et al., 2021).

A strategy for unloading the hamstring muscle and reducing injury risk is external support. Aldret et al. (2017) found that an elastic hamstring assistance device reduced participants’ subjective feelings of muscle soreness after long downhill running, yet with no significant effect on metabolic variables or performance. In the strive to develop a practical support aid for reducing hamstring-related injuries, this article presents a novel bracing concept—a unique dynamic spring brace. This brace utilizes the athlete’s end-range knee-flexion motions, during initial contact or late swings, to produce a force that posteriorly pulls the tibia (Veeck et al., 2023). These motions enable the brace to generate rapid and significant force during early knee-flexion, increase hamstring force, and improve the hamstring-quadriceps ratio. Indeed, lower hamstring-quadriceps ratios have been found to pose a major risk factor for hamstring injuries among soccer players (Veeck et al., 2023).

The aim of this study was threefold: (1) to test the effect of the dynamic brace on isolated hamstring-muscle performance in the field and to help identify the risk of injury among athletes, (2) to examine the effect of the brace on explosive performance in field tests, and (3) to evaluate the effect of the brace on the risk of knee-joint injuries.

## Methods

### Participants

A total of 13 healthy male basketball players took part in this study (age M = 16.2 ± 0.6 yrs; body height M = 1.76 ± 0.1 m; body mass M = 74 ± 3 kg). The following inclusion criteria were applied: the athlete had no prior hamstring injuries, had not undergone surgery on his lower extremities, and had no neurological / musculoskeletal condition that might hinder his ability to perform the physical tasks required in this study. The study was conducted in accordance with the Declaration of Helsinki, and was approved by the ethics committee of the Levinsky-Wingate Academic College (Wingate Campus), Netanya, Israel (reference number: 0163-23-TLV; date of approval: 03 September 2024). All participants submitted a signed consent form. For participants younger than 18 years old, one parent also submitted their informed written consent.

### Measures

#### The Novel Dynamic Hamstring Brace

**Figure 1:**
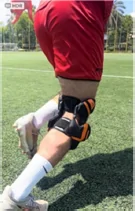
The dynamic knee brace.

As seen in Figure 1, the brace (325 g) had an upper strap that was attached to the athlete’s femur, and a lower strap that was attached around their tibia tuberosity. The brace had two customized springs that were calibrated, whereby 1-cm elongation produced a 20-N tension force; for each athlete, the spring tension was set based on their body mass and their subjective feelings and input, to ensure that the brace did not interfere with their natural movements during explosive performance. Arranged parallel to the posterior section of the brace, the springs were attached to two wires that went through a pulley to the tension mechanisms on the lateral and medial sides of the brace axis. When the knee was flexed, the springs elongated, following the motion that was created in both the brace axis and in the tension mechanism. This enabled 15–45° knee flexion. In turn, a force was produced, whereby the tibia was pulled posteriorly (LaPrade et al., 2017). As the flexion motion took place, the tension mechanisms gradually reduced the spring tension, enabling complete knee flexion.

#### Isolated Hamstring Muscle Tests

First, the end-range hamstring strength (ERHT) was measured for each participant using a hand-held dynamometer (Lafeytte, model 01165A). The participant was asked to lay on the physiotherapy bed in the supine position, and then pull the tested leg up to maximal hip flexion, using their own hands. The other leg remained in a neutral position. The examiner stood beside the bed, with one hand supporting the participants’ maximal hip flexion, the other hand holding the measuring device, with the elbow and the arm placed firmly by their side, to prevent movement of the measuring arm. The tester then placed the dynamometer on the participant’s calcaneus, while the participant was asked to maintain isometric knee flexion contraction for 3–5 s. This was repeated three times for each leg, with a 30-s rest interval between repetitions (Kristiansen et al., 2024). The best result out of the three attempts was documented for this study.

Next, the SLBT was conducted. For this test, the participant was instructed to lie on the ground, with one heel raised onto a 60-cm-high box, the other at an approximate 20° knee flexion. The participants were then instructed to cross their arms over the chest and push down using the raised heel, to lift their buttocks off the ground to extend their hip to 0°, and then back down again to the ground. They were asked to perform this exercise as many times as possible, without resting between repetitions. Consistent feedback was provided by the tester, to ensure that the correct technique was maintained. The height of their upward movement was measured using a one-meter scale, to maintain identical height throughout the repetitions within and between legs. Throughout this exercise, the non-working limb remained stationary, to prevent gaining momentum from swinging that leg. When a repetition was incorrectly performed, a warning was given to the participant. The test ended with the next incorrect performance (Freckleton et al., 2014). The number of valid repetitions was recorded for this study.

#### Performance Tests

To assess the participants’ anaerobic performance, the following tests were conducted, with two attempts for each test and a 1–3-min rest interval between them. The best result of the two attempts was recorded for subsequent analysis.

#### Horizontal Jump Tests

The aim of the 10-m and 20-m sprint tests was to evaluate the participants’ explosive horizontal power, with and without the brace. Participants began from a high standing position, with their dominant leg in front; they then sprinted forwards as fast as possible in a straight line. All attempts were measured in seconds using photocells (Witty Wireless Training Timer, Microgate, Bolzano, Italy) and recorded using a standard 2D video camera, to enable the manual counting of the number of strides taken by the dominant leg during the sprint (Erdman et al., 2024).

#### Vertical Jumps

The aim of these tests was to evaluate the participants’ explosive strength during vertical movements. For the squat jump (SJ), participants maintained a half-squat position for 3 s and then performed a powerful upwards jump, while continuously keeping their hands on their hips (Hughes et al., 2022). For the counter-movement jump (CMJ), participants stood with their hands on their hips, were then asked to bend their knees (to approximately 120°) as quickly as possible and immediately jump up as high as possible (Hughes et al., 2022). The Optojump Next (Microgate, Bolzano, Italy) was used to measure the vertical jumps. Finally, the frontal-plane protection angle (FPPA), i.e., the angle between the anterior superior iliac spine medical line of the knee and the medial malleolus, was measured, based on the video recordings of the CMJ tests. The knee maximal adduction angle during the loading phase was measured for the tested knee.

### Design and Procedures

The tests were randomly performed (research and control) on two different days during the same week, at the same time of the day. An exercise physiologist and a sports physiotherapist, both highly experienced, supervised the tests . The tests were performed after a 15-min dynamic warm-up. Each participant performed the tests twice: the first time while wearing the dynamic hamstring brace and the second time without. The brace was fitted for each participant with the help of the physiotherapist. Prior to performing each task, unlimited practice was allowed.

### Statistical Analysis

To ensure the robustness of the statistical evaluation, we conducted descriptive statistical analyses, a paired *t*-test, and the Wilcoxon matched-pairs test. Data were reported as mean ± standard deviation (SD). *A priori* power analysis was conducted using Power Analysis (G*Power version 3.1.9.7). Post-hoc power analysis was performed for the seven Wilcoxon paired comparisons. Cohen’s *d* effect sizes (Gignac et al., 2016) were used to assess the magnitude of the difference and were interpreted as follows: *d* < 0.20 was considered trivial, 0.21–0.50 small, 0.51–0.8 moderate, 0.81–1.10 large, and *d* > 1.10 very large. Significance was set at *p* < 0.05 for all statistical tests. Data were analyzed using SPSS (IBM Inc.) v.26.

## Results

Table 1 presents the mean and standard deviation (M ± SD) results for each test under both conditions and the effect size (ES); all tests exhibited large effect sizes and achieved relatively high statistical power (~0.96). All relevant performance data and statistical outcomes are summarized in Table 1.

**Table 1:** Performance test results.

Test	Control (M ± SD)	Brace (M ± SD)	Cohen's *d*	ES
ERHT (kg)	17.9 ± 5	18.7 ± 41	0.39	small
SLBT (rep)	23.5 ± 5	30 ± 4*	2.4	very large
10-m sprint (s) Dominant leg (strides)	1.88 ± 0.1 4.5 ± 0.4	2.01 ± 0.1* 4.4 ± 0.4	1.20	very large
20-m sprint (s) Dominant leg (strides)	3.32 ± 0.16 7.7 ± 0.8	3.38 ± 0.12 7.6 ± 1	0.2	small
SJ (cm)	39.4 ± 4	39.4 ± 4	0.3	small
CMJ (cm)	32.8 ± 4	31.94 ± 3	0.025	trivial
FPPA (degrees)	13.7 ± 5	7.3 ± 3*	2.43	very large

* p < 0.05; ERHT = end-range hamstring-strength; SLBT = single-leg bridge test; CMJ = counter-movement jump; SJ = squat jump; FPPA = frontal-plane protection angle; ES = effect size

**Figure 2:**
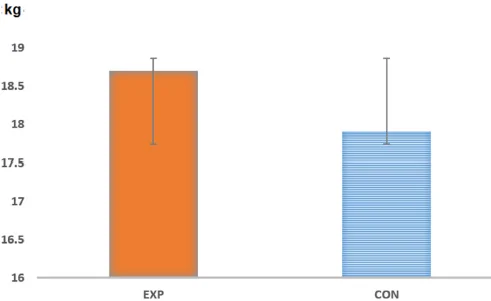
ERHT results: maximal force (kg). CON: control group (without the brace); EXP: experimental group (with the brace)

**Figure 3:**
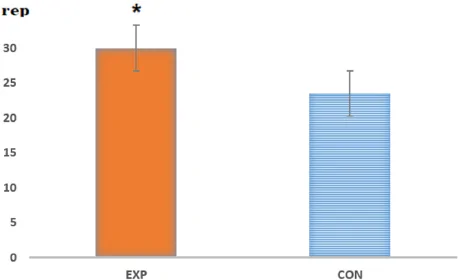
SLBT results (maximal number of repetitions). CON: control group (without the brace); EXP: experimental group (with the brace); rep: the number of repetitions; * p < 0.05

As seen in Figure 2, wearing the dynamic brace resulted in a significant improvement in the SLBT results under the experimental condition (i.e., with the brace) compared to the control one (M = 30 ± 4 repetitions vs. M = 23.5 ± 5 repetitions, respectively; *p* < 0.05) and demonstrated very large effect sizes (Cohen’s *d* = 2.4). Yet as seen in Figure 3, no significant increase was seen in ERHT when wearing the brace (M = 18.7 ± 4 kg vs. M = 17.9 ± 5 kg, respectively (*p* = 0.20) and demonstrated small effect sizes (Cohen’s *d* = 0.39).

**Figure 4:**
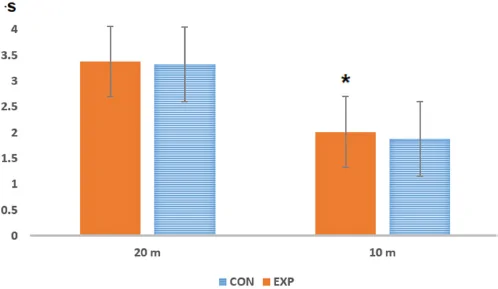
Horizontal explosive test results (s). CON: control group (without the brace); EXP: experimental group (with the brace); * p < 0.05

**Figure 5:**
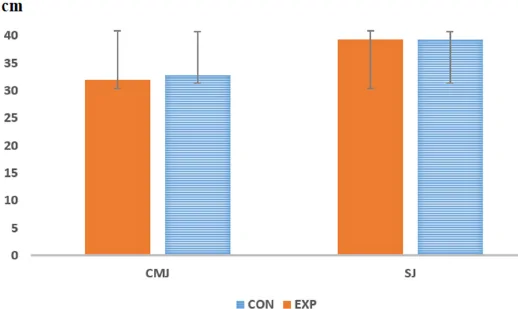
Vertical explosive test results (cm). CON: control group (without the brace); EXP: experimental group (with the brace)

In the 10-m sprint, wearing the brace resulted in a significantly slower time than without the brace (M = 2.01 ± 0.1 s and M = 1.88 ± 0.1 s, respectively; *p* < 0.05) and very large effect sizes were observed (Cohen’s *d* = 1.2). Moreover, no differences in the participants’ performance were seen in the 20-m sprint (M = 3.38 ± 0.12 s and M = 3.32 ± 0.16 s, respectively; *p* = 0.20) and small effect sizes were found (Cohen’s *d* = 0.2) (Figure 4). Additionally, no differences were seen between with and without the brace condition in the CMJ (M = 32.8 ± 4 cm vs. M = 31.9 ± 4 cm, respectively) and trivial effect sizes were observed (Cohen’s *d* = 0.025), nor in the SJ (M = 39.4 ± 4.0 cm vs. M = 39.4 ± 4.0 cm) with small effect sizes (Cohen’s *d* = 0.3) (Figure 5). Finally, when analyzing the 2D video recordings, a significant reduction was seen in the dynamic knee valgus, measured in the FPPA angle, without the brace (M = 7.3 ± 3° vs. M = 13.7 ± 5°, respectively; *p* < 0.05) and very large effect sizes were observed (Cohen’s *d* = 2.43).

## Discussion

This study aimed at examining the impact of a novel dynamic hamstring brace on athletic performance. The findings indicate that wearing the brace led to improved hamstring-muscle endurance when performing the SLBT. According to previous research (Freckleton et al., 2014), this field test is most predictive of incurring hamstring injuries. Moreover, although the brace required the athletes' motion for producing force, it did not improve overall performance in vertical jumps and only had a limited effect on short sprints of 10 m. The effects of the brace on the dynamic knee valgus are in line with previous literature. These findings are of clinical importance, due to the widespread incidence of hamstring injuries in sports.

A large percentage of hamstring injuries (about 70%) have been found to occur during sprinting (Edouard et al., 2023), especially during the late swing and initial stance stages, when large passive torque at the knee and hip joints works together to extend the hamstring muscles. This indicates that the hip extensors and knee flexors play a major role in sprint running (Silvers-Granelli et al., 2021).

Due to the seriousness of this injury, an emphasis has been placed on developing hamstring field tests, based on various hip and knee positions. The two main tests are the SLBT, which examines the eccentric endurance muscle capability (Freckleton et al., 2014) and the ERHT test, which assesses the dynamic capability of the muscle to produce rapid maximal force (Kristiansen et al., 2024). Yet, the SLBT was found to have greater predictive value for identifying at-risk athletes (Freckleton et al., 2011), especially in individuals with lower physical fitness (Mahnič et al., 2021)—as muscle fatigue is a major risk factor of hamstring injuries (Ekstrand et al., 2023b). In contrast, the ERHT test has been found to strongly correlate with peak muscle force, measured through isokinetic tests, yet with little predictive value regarding athletes' risk of injury (Miralles-Iborra et al., 2023).

The results of the current study indicate that the dynamic brace significantly increases the hamstring’s eccentric and passive force and muscle endurance, yet not its maximal force. This could be due to each spring force, which reaches a maximum of 20 N; this may not be adequate for increasing maximal force. However, the additional force that was provided by the springs did improve muscle endurance and resistance to fatigue, thereby potentially reducing injury risk.

The results of the isolated hamstring test explain the effect of the brace on the horizontal and vertical performance tests. The slower time achieved in the 10-m sprint with the brace was not due to the additional weight of the brace (325 g), as this did not lead to a significant slowdown in the 20-m run or a decrease in the vertical jump. This increased time duration could be explained by the acceleration phase (von Lieres und Wilkau et al., 2018), with increased leg movements comprised of shorter strides (performing 5 strides during the 10-m test compared to 3 strides in the dominant leg measured between the 10–20-m section. We can therefore assume that the small change in the hamstring-quadriceps ratio due to the brace’s action, i.e., posteriorly pulling the tibia at 15–30° during a higher rate of strides, could reduce athletes' acceleration performance. In the current study, in the 20-m sprint, when stride frequency decreased, the effect of the dynamic brace was not significant.

Additionally, the lack of effect of the brace on vertical performance could be explained by the starting position, more specifically the knee-flexion degrees: 87–107° in the CMJ and 70° in the SJ (Pérez-Castilla et al., 2021). As such, larger knee flexion may enable the quadriceps to develop increased force during acceleration.

Although this study was conducted among youth basketball players, prospective research has shown that the prevalence of hamstring injuries in this age group is increasing and approaching levels observed in adult athletes (Valle et al., 2018). This finding suggests that the use of a brace may also offer potential benefits for adult athletes recovering from hamstring injuries.

Finally, in line with previous studies, wearing the brace significantly reduced the dynamic knee valgus angle compared to the control condition, which may have resulted from the static structure of the brace, as shown in previous studies (Gentile et al., 2021).

### Limitations and Future Research Directions

This study offers important insights regarding the novel dynamic knee brace. Yet a number of research limitations should be addressed. First, all performance tests that were applied in this study were field tests. Also conducting laboratory tests, such as isokinetic muscle tests, might provide additional data on the hamstring-quadriceps ratio at different velocities and angles. Moreover, all participants were healthy young male individuals. As such, generalization of the findings should be conducted with caution.

Performing the study on additional demographic groups and fields of sports, and on athletes who have recovered from a hamstring injury would provide additional insights. Finally, further research should evaluate the effect of continuous brace use and more precise stabilization force adjustments on performance variables and on reinjury rates—during hamstring rehabilitation and shortly after returning to play.

## Conclusions

The dynamic hamstring brace examined in this study was able to be adjusted to the participants’ physical variables and subjective comfort, thereby improving hamstring-muscle endurance in vulnerable positions, with the potential of reducing muscle injuries. Based on these results, the dynamic brace can be used during rehabilitation and upon returning to full athletic activities, possibly also reducing recurring injuries.
